# Supplementary material for: Advantages in Wound Healing Process in Female Mice Require Upregulation A2A-Mediated Angiogenesis under the Stimulation of 17β-Estradiol
Source: Int J Mol Sci. 2020 Sep 28;21(19):7145. doi: 10.3390/ijms21197145 (PMC7583763; doi:10.3390/ijms21197145)
Supplement: Supplementary file 1 [file ijms-21-07145-s001.pdf]

Advantages in Wound Healing Process in Female Mice Require Upregulation A_{2A}-mediated Angiogenesis under the Stimulation of 17β-estradiol

Felipe Troncoso [1,2], Kurt Herlitz [1], Jesenia Acurio [1,3], Claudio Aguayo [3,4], Katherine Guevara [1], Fidel Ovidio Castro [5], Alejandro S. Godoy [3,6,7], Sebastian San Martin [3,8] and Carlos Escudero [1,3,*]

[1] Vascular Physiology Laboratory, Department of Basic Sciences, Universidad del Bío-Bío, 3780000 Chillán, Chile; fetronc@gmail.com (F.T.); kherlitz@gmail.com (K.H.); jeseniacurio2@gmail.com (J.A.); kattyguevarae4@gmail.com (K.G.)
[2] Programa de Doctorado en Ciencias Veterinarias, Universidad de Concepción, 3780000, Chillán, Chile
[3] Group of Research and Innovation in Vascular Health (GRIVAS Health), 3780000 Chillán, Chile; alejandro.godoy@uss.cl (A.S.G.); sebastian.sanmartin@uv.cl (S.S.M.)
[4] Department of Clinical Biochemistry and Immunology, Faculty of Pharmacy, University of Concepción, 4030000 Concepción, Chile
[5] Department of Animal Science, Faculty of Veterinary Sciences, Universidad de Concepcion, 3780000 Chillán, Chile; fidcastro@udec.cl
[6] Department of Urology, Roswell Park Comprehensive Cancer Center, Buffalo, NY 14263, USA
[7] Centro de Biología Celular y Biomedicina (CEBICEM), Universidad San Sebastián, 8320000 Santiago, Chile
[8] Biomedical Research Centre, School of Medicine, Universidad de Valparaíso, 2340000 Valparaíso, Chile
* Correspondence: cescudero@ubiobio.cl; Tel.: +56-42-2463256 or +56-9-65655127

Running title: A_{2A} and estrogens.

*Correspondence: Carlos Escudero, MD PhD
Vascular Physiology Laboratory
Group of Research and Innovation in Vascular Health
Basic Sciences Department
Faculty of Sciences
Universidad del Bio-Bio
Chillán, Chile
Phone: 56-42-2463256 / Mobile: 56-9-65655127
cescudero@ubiobio.cl

Word count: 5210 (main text)

Financial disclosure: none

Conflict of interest: none

Number of tables: 2 Number of figures: 7. Number of references: 38. Supplementary information: Table S1. Figure S1 and S2

Key code: Angiogenesis, Endothelium, Physiology, Vascular Biology.

Abbreviations: A_{2A} deficient mice, A_{2A} KO; Estrogen receptor alpha, ERα; Estrogen receptor beta ER-β; Human umbilical vein endothelial cell, HUVEC; Mice pulmonary endothelial cells, mPEC; Microvascular endothelial cell line, HMEC-1; Platelet endothelial cell adhesion molecule 1, PECAM-1 or CD31; Vascular endothelial growth factor, VEGF.

Methodology

Laser Doppler Perfusion.

Tissue perfusion was analyzed in the wounded area at day four (D4) after injury in female and male WT or A_{2A}KO mice (3 per group) using a Speckle Doppler Perfusion Imager "PeriCAm PSI-HR system" (Perimed Ltd., Stockholm, Sweden). Blood flow was recorded for 5 minutes on D4 after injury in the healing area and normalized to a low perfusion area located less than 1 centimeter away in the same animal (regions of interest, ROIs). Time of interest (TOI) was defined between 2 and 3.2 min of records in order to avoid artifacts during recording. The images were blindly analyzed by two observers.

Vessel counting in the wounded area

For histological analysis, skin tissue extraction was performed after euthanasia at D4. Day 4 was defined considering previous evidences of faster re-epithelialization, increased matrix deposition, high fibroblast density and vascularity in the granulation tissue in mice treated with A_{2A} agonist topically (Montesinos et al., 2002). Extracted tissues were fixed by immersion in 4% (v/v) formalin in PBS for 4 days to 4°C, after that tissues were embedded in paraffin. Six-micron-thick sections were stained using hematoxylin and eosin and analyzed under light microscopy. Photos were taken at 10X magnification and used for measuring the number of blood vessels in the dermis by two independent observers.

Table S1. Sequence of the used primers

Gene	Primers sequence	Tm	Expected size
A_1	F 5” CGG-GAT-CCT-ACA-TCT-CGG-CCT-TCC-AGG-3”	58	219
	R 5” GGA-ATT-CAG-TAG-GTC-TGT-GGC-CCA-ATG-3”		
A_{2A}	F 5” AGC-CAG-GGG-TTA-CAT-CTG-TG-3”	56	177
	R 5” AGA-CAA-TCG-GCT-GCT-CTG-AT-3”		
A_{2B}	F 5”-CGG-GAT-CCT-TTC-ACG-GCT-GCC-TCT-TC-3”	58	271
	R 5”-GGA-ATT-CCA-TCC-CCC-AGT-TCT-GTG-C-3”		
A_3	F 5” CGG-GAT-CCC-GTT-CCG-TGG-TCA-GTT-TG-3”	56	363
	R 5” GGA-ATT-CGC-AGG-CGT-AGA-CAA-TAG-G-3”		
Neomycin (NEO)	F 5” AGA-CAA-TCG-GCT-GCT-CTG-AT-3”	56	618
	R 5” CAA-GCT-CTT-CAG-CAA-TAT-CAC-G 3”		
mlp37	F 5” TCT-TCC-GGT-CTC-TTT-GGC-CT	56	297
	R 3” CTT-GGG-TTT-CGG-CGT-TGT-TC-5”		
Jarid 1c/1d	F 5′ CTG-AAG-CTT-TTG-GCT-TTG-AG-3”	58	331
	R 5′ CCA-CTG-CCA-AAT-TCT-TTG-G-3		

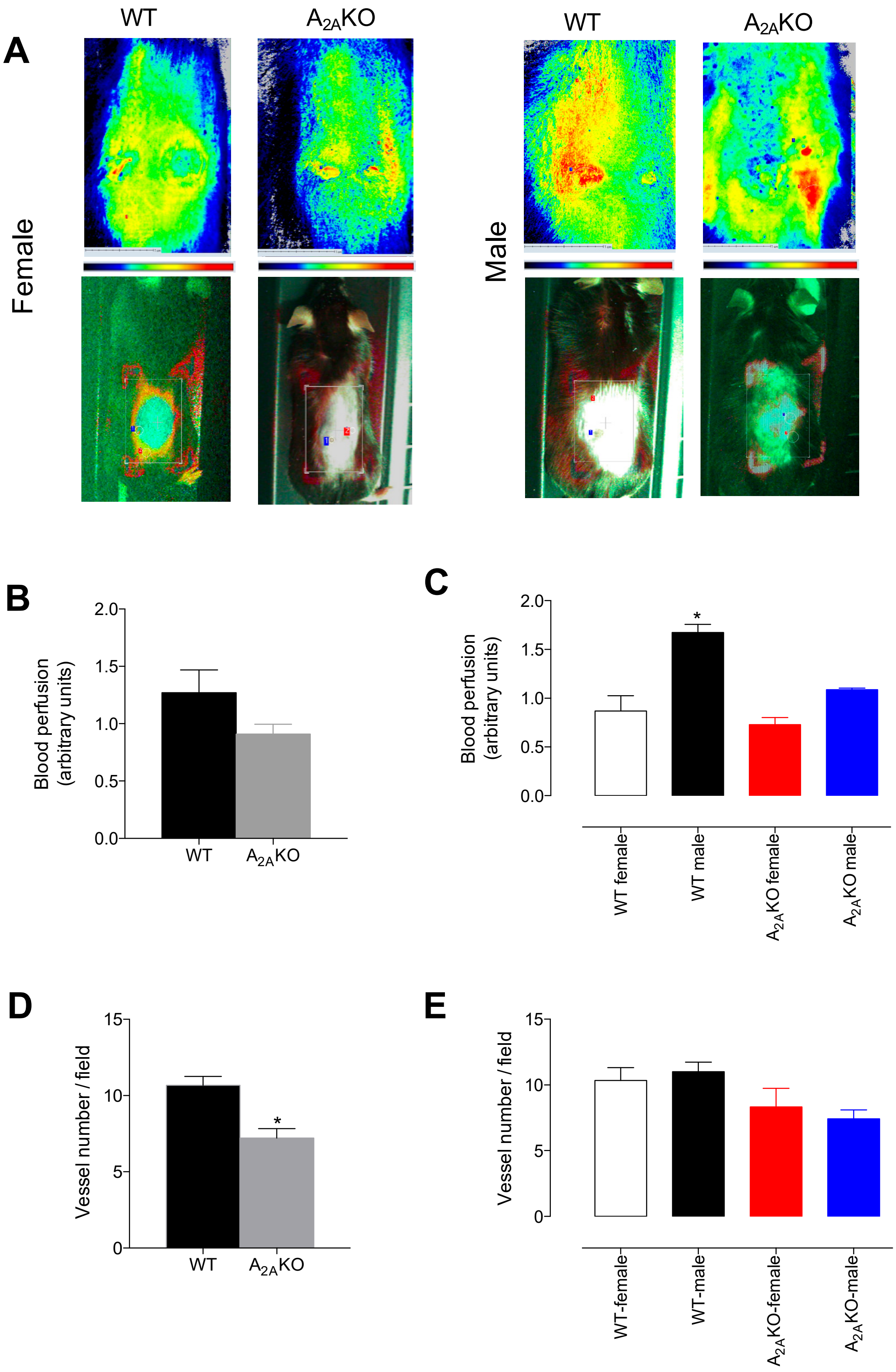
A
WT
A2AKO
Female
WT
A2AKO
Male
B
Blood perfusion (arbitrary units)
2.0
1.5
1.0
0.5
0.0
WT
A2AKO
C
Blood perfusion (arbitrary units)
2.0
1.5
1.0
0.5
0.0
*
WT female
WT male
A2AKO female
A2AKO male
D
Vessel number / field
15
10
5
0
*
WT
A2AKO
E
Vessel number / field
15
10
5
0
WT-female
WT-male
A2AKO-female
A2AKO-male

Figure S1. Blood perfusion and vessel count in wild type and A_{2A}KO mice: **A)** Representative images of blood perfusion using Laser Doppler in female and male wild type (WT) and A_{2A} deficient mice (A_{2A}KO) at day 4 (D4) after injury. **B)** Blood perfusion in arbitrary units in the whole group of WT and A_{2A}KO mice. **C)** Blood perfusion in male and female WT and A_{2A}KO mice. Pseudocolor scale represents larger perfusion (red) to no perfusion (blue). Photos show the experimental specimen (mice) and the location in which the perfusion of the wounded area (red dot) or the peripheral area (blue dot) were analyzed. **D)** Quantification of the number of blood vessel in the dermis of the wounded area at day 4 after injury considering the whole group; or **E)** Sex-differences in both WT and A_{2A}KO mice. In C, *$P<0.05$ versus female WT mice. In D, *$P<0.05$ versus WT. Values were expressed as mean ± SEM. n=3-4 per group.

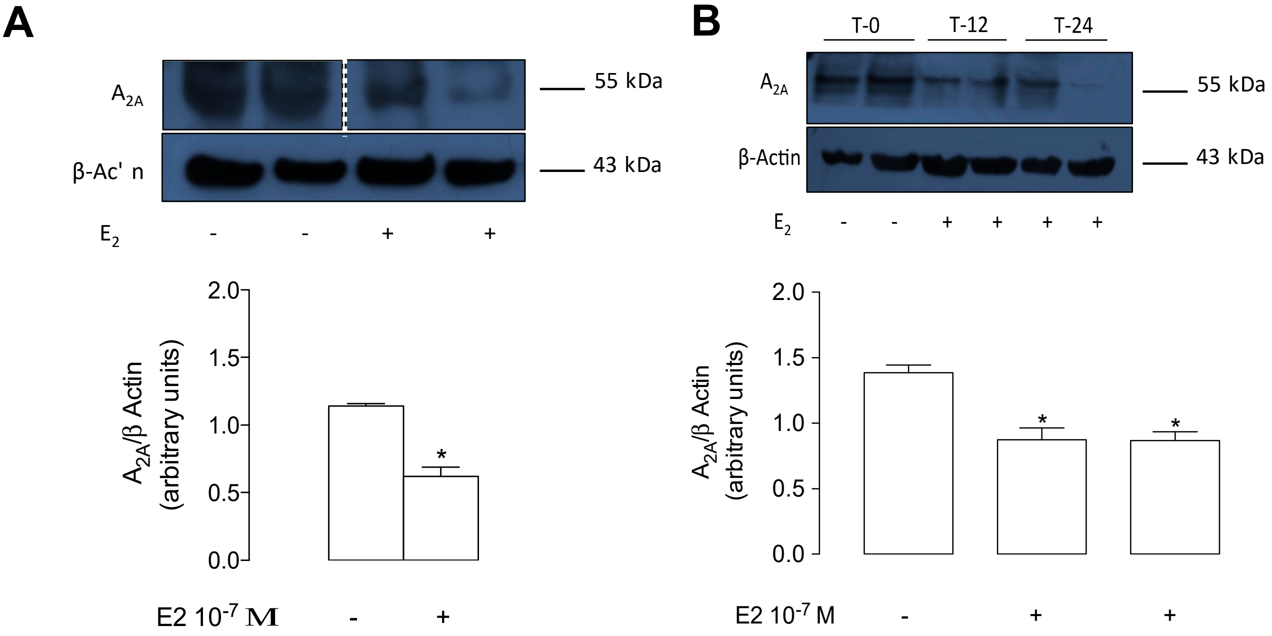

Figure S2. 17β-estradiol down regulated the protein expression of A_{2A} in female endothelial cells. A) Western blot analysis of A_{2A} protein levels on female mPEC, or **B)** Female human umbilical vein endothelial cells (HUVEC) in absence (-, control) or presence (+) of 17β-estradiol (10^{-7}M x 12 h). Dashed line in A indicate that proteins were run in different gels. *P<0.05 vs control. Values were expressed as mean ± SEM. n = 3-4 per group.

References

Montesinos, M.C., Desai, A., Chen, J.F., Yee, H., Schwarzschild, M.A., Fink, J.S., et al. (2002). Adenosine promotes wound healing and mediates angiogenesis in response to tissue injury via occupancy of A(2A) receptors. *Am J Pathol* 160(6), 2009-2018. doi: 10.1016/S0002-9440(10)61151-0.
